# Effects of Soil Moisture on the Temperature Sensitivity of Soil Heterotrophic Respiration: A Laboratory Incubation Study

**DOI:** 10.1371/journal.pone.0092531

**Published:** 2014-03-19

**Authors:** Weiping Zhou, Dafeng Hui, Weijun Shen

**Affiliations:** 1 Key laboratory of Vegetation Restoration and Management of Degraded Ecosystems, South China Botanical Garden, Chinese Academy of Sciences, Guangzhou, China; 2 Graduate University of the Chinese Academy of Sciences, Beijing, PR China; 3 Department of Biological Sciences, Tennessee State University, Nashville, Tennessee, United States of America; North Carolina State University, United States of America

## Abstract

The temperature sensitivity (Q_10_) of soil heterotrophic respiration (R_h_) is an important ecological model parameter and may vary with temperature and moisture. While Q_10_ generally decreases with increasing temperature, the moisture effects on Q_10_ have been controversial. To address this, we conducted a 90-day laboratory incubation experiment using a subtropical forest soil with a full factorial combination of five moisture levels (20%, 40%, 60%, 80%, and 100% water holding capacity - WHC) and five temperature levels (10, 17, 24, 31, and 38°C). Under each moisture treatment, R_h_ was measured several times for each temperature treatment to derive Q_10_ based on the exponential relationships between R_h_ and temperature. Microbial biomass carbon (MBC), microbial community structure and soil nutrients were also measured several times to detect their potential contributions to the moisture-induced Q_10_ variation. We found that Q_10_ was significantly lower at lower moisture levels (60%, 40% and 20% WHC) than at higher moisture level (80% WHC) during the early stage of the incubation, but became significantly higher at 20%WHC than at 60% WHC and not significantly different from the other three moisture levels during the late stage of incubation. In contrast, soil R_h_ had the highest value at 60% WHC and the lowest at 20% WHC throughout the whole incubation period. Variations of Q_10_ were significantly associated with MBC during the early stages of incubation, but with the fungi-to-bacteria ratio during the later stages, suggesting that changes in microbial biomass and community structure are related to the moisture-induced Q_10_ changes. This study implies that global warming’s impacts on soil CO_2_ emission may depend upon soil moisture conditions. With the same temperature rise, wetter soils may emit more CO_2_ into the atmosphere via heterotrophic respiration.

## Introduction

Temperature sensitivity of soil respiration, usually termed as Q_10_, is defined as the increase of soil respiration rate by a 10°C rise in temperature [1]. Q_10_ has been considered an important model parameter in predicting terrestrial ecosystem carbon cycle and feedback to climate warming [2]. In the past several decades, Q_10_ has been investigated extensively, particularly through field-observed soil respiration and environmental factor data [3], [4]. It has been found that Q_10_ is not a constant of 2, but varies with vegetation and edaphic conditions such as temperature, moisture, and substrate availability [2]. As global temperature continues to rise [5], it is of paramount importance to understand how Q_10_ is influenced by these factors individually and interactively. Since under field conditions, effects of soil temperature and moisture on Q_10_ are often confounded with each other and with other factors, laboratory incubation has the advantage of deriving the primary and interactive effects of the environmental factors on Q_10_.

Many studies have demonstrated that Q_10_ can be influenced by a variety of biological and environmental factors [1], [6], [7]. Soil temperature itself has been found to have a negative correlation with Q_10_. For example, at lower temperature regions (e.g., tundra), Q_10_ tends to be higher than the estimates at warmer temperature regions (e.g., warm desert) [8]. A manipulated warming experiment also demonstrates that Q_10_ is significantly lower at high temperature treatments than at the low temperature control [1]. Thus, the temperature effects on Q_10_ have been generally consistent; i.e., Q_10_ decreases with increasing temperature. However, the effects of other factors such as soil moisture on Q_10_ have been less certain and deserve more research.

Soil moisture plays a critical role in soil respiration and may have a significant impact on Q_10_ [9]–[11]. The basic concepts and mechanisms of soil moisture on soil respiration have been discussed by many researchers [12]–[14]. The optimum soil moisture for soil respiration is frequently found at intermediate levels, above or below which soil respiration decreases [15]. At the optimum soil moisture, the macropore spaces are filled with adequate amounts of air and water which can facilitate the diffusion of both oxygen and soluble substrates [16]. In very wet soils oxygen limitation occurs, and in very dry soils the movement of soluble substrates via water films is restricted. Although the mechanistic understanding on the effects of soil moisture on R_h_ has been largely advanced, its influence on the Q_10_ of R_h_ is still inconclusive. For example, Wang et al. [17] reported that Q_10_ increased with soil moisture until reaching a threshold, and then declined in six temperate forests of China. Carlyle and Than [18] showed that soil moisture limited the Q_10_ of soil respiration beneath a *pinus radiata* stand in south-eastern Australia. But Reichstein et al. [19] found that Q_10_ was insensitive to the drying of a spruce forest soil. The inconsistency of soil moisture effects on Q_10_ is probably due to the confounding influences of different environmental factors under field conditions. One recent incubation study showed that soil moisture indeed influenced Q_10_ and the moisture-Q_10_ relationship differed between soils obtained at different topographic positions [20], but the underlying mechanisms remained unclear.

Effects of soil moisture on Q_10_ may be ascribed to changes in microbial biomass and community structure, and the physical and chemical properties of the soil [7], [21]. Changes in soil moisture can affect the composition and function of soil microbial community due to differences in drought tolerance among taxonomic and functional groups of microorganisms [22]. For example, fungi can survive drought stress better than bacteria due to their ability to grow at lower matric potentials [23], [24]. Soil moisture can also affect the quantity of soil microbial biomass carbon (MBC) and dissolved organic carbon (DOC) [25], [26]. Despite a general understanding of the above processes, whether soil moisture effects on Q_10_ can be related to its influences on soil properties such as MBC, DOC, nutrient availability, and microbial community structure is still in active debate.

In this study, we investigated soil moisture effects on Q_10_ by incubating a subtropical forest soil under five temperature levels and five moisture levels over 90 days. Soil R_h_ and other properties such as MBC and DOC, nitrogen and phosphorous contents, and microbial community phospholipid fatty acids (PLFAs) were measured several times during the incubation period. Our objectives were first to analyze how changes in soil moisture influenced Q_10_, and second to explore whether the moisture effects on Q_10_ could be related to its impacts on the soil microbial and chemical properties measured.

## Materials and Methods

### Ethics Statement

Soils were sampled from a study site that is maintained by the South China Botanical Garden, Chinese Academy of Sciences. All necessary permits were obtained for the described study. This study did not involve endangered or protected species.

### Site Description

Incubation soils used in this study were collected from an evergreen broadleaved forest stand at the Heshan Hilly Land Interdisciplinary Experimental Station (22°34′N, 112°50′E) in Guangdong Province of China. The region has a subtropical humid monsoon climate with apparent dry and wet seasons. The wet season starts in April and ends in October, and the dry season begins in November and lasts through March of the following year. The mean annual precipitation and temperature are 1700 mm and 21.7°C, respectively. The forest stand is 29 years old and mainly dominated by native tree species (*Schima superba* and *Michelia macclurei*) with an average height of 15 m and an average diameter at breast height (DBH) of 30 cm. The soil is categorized as Oxisols based on the US Soil Classification System [27], [28], with a bulk density of 1.4 g cm^−3^, total organic carbon (TOC) of 2.80%, total nitrogen (TN) of 0.15%, and total phosphorous (TP) of 0.02% at the depth of 0–20 cm.

### Incubation Experimental Design

In the field, four sampling areas, with a distance of at least 10 m between each, were selected to collect the incubation soils. In each area, five sampling sites (20×10 cm^2^) were randomly selected and sampled to the depth of 20 cm. These five random samples were homogenized to form a composite sample. Before sampling, the uppermost layer of litter with visible un-decomposed materials was excluded. We had four composite samples as four experimental replicates, each one weighing about 50 kg in fresh weight. All soil samples were transported to the laboratory and passed through a 2 mm sieve with apparent plant roots and stones being removed.

To investigate soil moisture effects on the temperature sensitivity (Q_10_) of soil R_h_, we used five soil moisture levels: 20%, 40%, 60%, 80%, and 100% water holding capacity (WHC). For each moisture level, soils were incubated under five temperature levels: 10, 17, 24, 31, and 38°C. A full factorial combination of the two factors and five levels for each factor produced 25 experimental treatments. Each treatment had four replicates from the four composite samples. Each replicate further had 6 duplicates, with one duplicate for measuring R_h_ and the other 5 for destructive sampling. As a result, we had 600 incubation soil samples in total with 120 ( = 5 moisture levels × 4 replicates × 6 duplicates) in each of the five static temperature incubators (RXZ-600B, Southeast Instrument Co., Ltd., Ningbo, China). The temperature and relative humidity deviations of the incubators are ±1.5°C and ±7%, respectively. Each air-dried incubation soil sample (equivalent to 50 g of oven-dried soil) was added to each triangle flask and its soil water content was adjusted to the corresponding soil moisture level by adding deionized water. The flasks were covered by rubber stoppers with small holes to reduce water loss via evaporation and maintain gas exchange. In order to maintain constant soil moisture levels, water loss was checked and corrected weekly by weighing each flask and adding water as necessary. At most, 1 ml of water (equivalent to 3.3% of changes in 100% WHC) was added every week to the flask at the temperature level of 38°C.

### Measurements of Soil R_h_, Microbial and Chemical Properties

The incubation experiment lasted for 90 days. Soil R_h_ rates were measured using the Li-6262 Infrared Gas Analyzer (Li-Cor Inc., Lincoln, NE) on days 1, 2, 3, 4, 6, 7, 13, 18, 27, 34, 41, 53, 62, 74, and 90. Electrical fans blowing air into each incubator for 30 min every four days were used to maintain an aerobic incubation environment. Before R_h_ measuring, each triangle flask was ventilated for 3 minutes to minimize gas accumulation in the headspace. After ventilation, another type of rubber stoppers with two plastic tubes for gas inlet and outlet was used to seal the flask and the tubes were connected to Li-6262 for measuring headspace CO_2_ concentration. The CO_2_ concentration in the headspace was recorded every second for 2 minutes and R_h_ rate was calculated using the linear portion of the response curve of CO_2_ concentration versus time [29]. At each moisture level and each measurement day, Q_10_ was calculated by fitting an exponential function to the measured R_h_ against the 5 temperature levels:
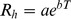
(1)where R_h_ is the measured soil heterotrophic respiration rate (μg g^−1^ oven dried soil h^−1^), T is the incubation temperature (°C), a and b are the fitted model parameters. Q_10_ is calculated individually for each of the four replicates using the following equation:



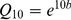
(2)As intrinsic Q_10_ is based on the kinetic reactions of molecular structure changes with temperature [2], the Q_10_ of this study is the apparent temperature sensitivity.

For measuring MBC, DOC, total organic carbon (TOC), total nitrogen (TN), total phosphorous (TP), inorganic N (NH_4_^+^ and NO_3_^−^), and PLFAs, three flasks (or replicates) of each treatment were harvested on days 7, 30, and 90. Part of the soil in each flask was collected and stored at −20°C for later analysis of PLFAs. The remaining soil was used to analyze microbial biomass and chemical properties. It is noted here that TOC, TN, and TP were only analyzed for the samples harvested on days 7 and 90.

Soil MBC was measured using the modified fumigation-extraction method [30]. MBC was calculated as the difference in extractable C concentrations between the fumigated and un-fumigated samples divided by a K_EC_ factor of 0.38 [30]. The extractable C concentrations of un-fumigated samples were the soil DOC [31]. Soil NH_4_^+^ and NO_3_^−^ were measured by the method proposed in Dorich and Nelson [32]. Soil TOC was measured using the potassium bichromate-concentrated sulphuric acid heating method. Soil TN and TP were measured using the Kjeldahl resolution Auto Flow Injection method. Microbial community PLFAs were analyzed according to Bossio and Scow [33]. Concentrations of each PLFA were calculated relative to the 19∶0 internal standard concentrations. 15∶0, i15∶0, a15∶0, i16∶0, 16∶1w7c, 17∶0, i17∶0, a17∶0, cy17∶0, 18∶1w7c, 19∶0 cyclow8c were selected as bacterial biomarkers and 18∶2w6, 9c were selected as fungal biomarkers [34], [35].

### Statistical Analysis

Repeated-measures ANOVA was employed to determine the effects of sampling time and soil moisture on Q_10_ and R_h_ on the 15 measuring days. One-way ANOVA was used to analyze soil moisture effects on Q_10_ for days 7, 30, and 90; Tukey’s HSD multiple comparison method was used to test Q_10_ differences among soil moisture levels. Regression analysis was used to derive the relationships between R_h_ and temperature, and between Q_10_ (or R_h_) and incubation time. Pearson correlation analysis was applied to detect the potential contributions of soil microbial and chemical properties on the Q_10_ variations with moisture. All these statistical analyses were performed using SPSS software 16.0 (SPSS Inc., Chicago, IL).

## Results

### Relationships between Soil R_h_ and Temperature under Different Soil Moisture Treatments

**Figure 1:**
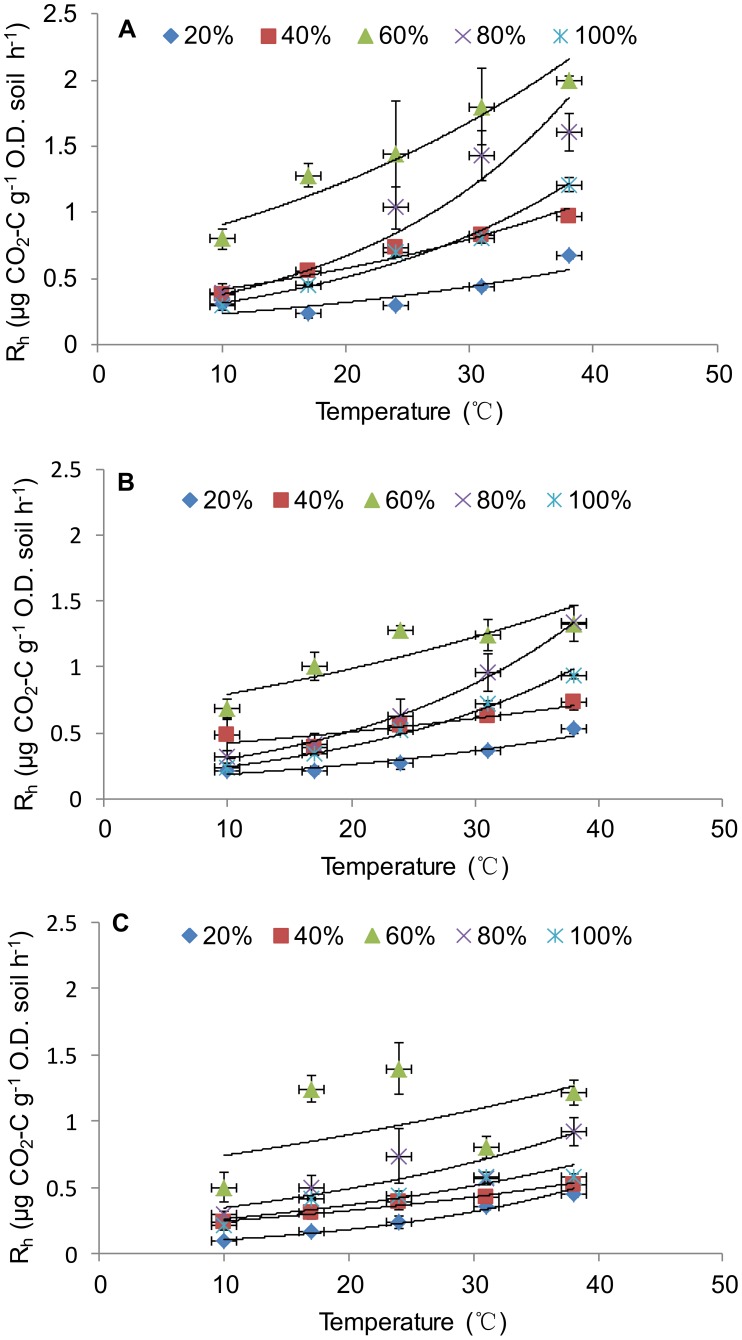
Responses of soil heterotrophic respiration (R_h_) to changes in soil temperature after 7 (A), 30 (B), and 90 (C) days of incubation. Each data point is the mean of four replicates under each soil moisture treatment. Error bars represent standard errors (n = 4).

Soil R_h_ varied significantly with temperature and incubation time under all the 5 moisture treatments (Table 1). R_h_ responses to temperature changes on days 7, 30, and 90 were displayed to represent the early, middle, and late incubation stages (Fig. 1). Throughout the whole incubation period, soil R_h_ was highest at 60% WHC, lowest at 20% WHC, and intermediate at the other moisture levels (Fig. 1). Among the three measurement days, soil R_h_ had the highest values on day 7, especially at 60% and 80% WHC, compared to those on days 30 and 90. Soil R_h_ declined with incubation time, declining in smaller magnitudes at 20% WHC and 100% WHC.

**Table 1:** Repeated-measures ANOVA for the temperature and time effects on R_h_ under different soil moisture treatments.

Moisture (a %WHC)	Temperature (°C)		Time (day)		Temperature×Time	
	F_(4, 15)_	P	F_(14, 210)_	P	F_(56, 210)_	P
20%	36.96	<0.01	26.59	<0.01	5.46	<0.01
40%	35.77	<0.01	50.03	<0.01	3.88	<0.01
60%	10.90	<0.01	45.26	<0.01	6.63	<0.01
80%	12.24	<0.01	52.44	<0.01	6.47	<0.01
100%	139.15	<0.01	159.63	<0.01	17.60	<0.01

a %WHC: percent of water holding capacity.

The temperature response of soil R_h_ could be well fitted using the exponential model for each soil moisture treatment and measurement day (Fig. 1). Model parameters for the three representative days are presented in Table 2. All models were significant with the coefficient of determination (R^2^) ranging from 0.72 to 0.98, except for (R^2^ = 0.25) at 60% WHC on day 90. Estimated basal soil respiration (i.e. parameter a) ranged from 0.06 to 0.66 and was higher at 60% WHC than at the other soil moisture treatments. The exponent (i.e. parameter b) ranged from 0.02 to 0.05 and was also lowest (0.019) at 60% WHC on day 90.

**Table 2:** Regression equations of heterotrophic respiration (R_h_) with temperature under different moisture treatments.

Soil moisture (a %WHC)	Day 7	Day 30	Day 90
20	b R_h_ = 0.1684e^0.0318T^	R_h_ = 0.1333e^0.0335T^	R_h_ = 0.062e^0.0546T^
	c R^2^ = 0.72*	R^2^ = 0.92**	R^2^ = 0.98**
40	R_h_ = 0.3014e^0.0324T^	R_h_ = 0.3526e^0.0183T^	R_h_ = 0.1896e^0.0273T^
	R^2^ = 0.95**	R^2^ = 0.72*	R^2^ = 0.97**
60	R_h_ = 0.6639e^0.031T^	R_h_ = 0.6382e^0.0218T^	R_h_ = 0.6148e^0.019T^
	R^2^ = 0.92**	R^2^ = 0.78*	R^2^ = 0.25
80	R_h_ = 0.2136e^0.057T^	R_h_ = 0.1774e^0.0532T^	R_h_ = 0.2484e^0.0342T^
	R^2^ = 0.92**	R^2^ = 0.99**	R^2^ = 0.77*
100	R_h_ = 0.1926e^0.0485T^	R_h_ = 0.1469e^0.0501T^	R_h_ = 0.19e^0.0333T^
	R^2^ = 0.98**	R^2^ = 0.99**	R^2^ = 0.81*

a %WHC: percent of water holding capacity.

b R_h_ represents soil heterotrophic respiration rate and T represents temperature.

c R^2^ is the coefficient of determination; * and ** indicate significance at P≤0.05 and P≤0.01, respectively.

### Changes of Q_10_ with Incubation Time under Different Soil Moisture Treatments

**Figure 2:**
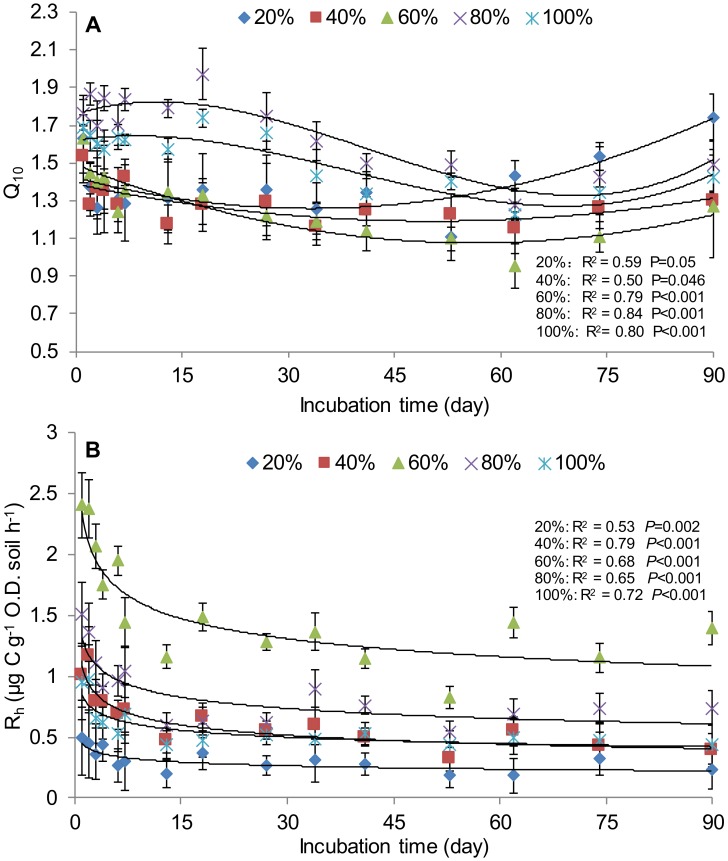
Changes of Q_10_ and R_h_ with incubation time at different soil moisture treatments. The Q_10_ regression functions are quadratic for 20%, 40% and 60% WHC and cubic for 80% and 100% WHC. Error bars (n = 4) represent standard deviations. R^2^ is the coefficient of determination. P is the significance level.

Under all the 5 moisture treatments, Q_10_ values ranged from 0.95 to 1.97 and varied markedly with incubation time. Q_10_ was higher in the beginning of the incubation, and declined with time to around day 60, then increased slightly to the end of the experiment (Fig. 2A). Among different soil moisture treatments, Q_10_ at 80% and 100% WHC was higher than at the other soil moisture treatments. Q_10_ at 60% WHC had the lowest value, especially after day 30. Q_10_ at 20% WHC was among the lowest in the beginning of the incubation, but increased with incubation time and had the highest values at the end of the experiment (Fig. 2A).

To better show the Q_10_ variation pattern with incubation time, polynomial regression models were used to fit Q_10_ with incubation time. Findings indicate that a quadratic regression model could fit Q_10_ well at 20%, 40% and 60% WHC with R^2^≥0.50 (Fig. 2A), while a cubic regression model should be applied at 80% and 100% WHC (R^2^≥0.80; Fig. 2A). Further inspection revealed the days on which the minimum and maximum Q_10_ appeared. At 20%, 40%, and 60% WHC, the lowest Q_10_ appeared on days 53, 62, and 62 with their values being 1.11, 1.15, and 0.95, respectively. At 80% and 100% WHC, Q_10_ showed the highest values of 1.94 and 1.74 on day 18, and the lowest values of 1.28 and 1.22 on day 62, respectively.

In contrast to the Q_10_ dynamics, R_h_ was always higher at 60% WHC, lower at 20% WHC and somewhere in between at the other three moisture levels (80%, 40%, and 100% WHC) (Fig. 2B). It is noted here that the R_h_ data shown in Fig. 2B are only those under the incubation temperature of 24°C, because under the other temperature treatments the general variation patterns of R_h_ with moisture and incubation time were similar. In our incubation experiment, the declination of R_h_ with incubation time could be best fitted using a power law decay function, with the coefficient of determination ranging from 0.53 to 0.78 (Fig. 2B).

### Effects of Soil Moisture on Q_10_

**Figure 3:**
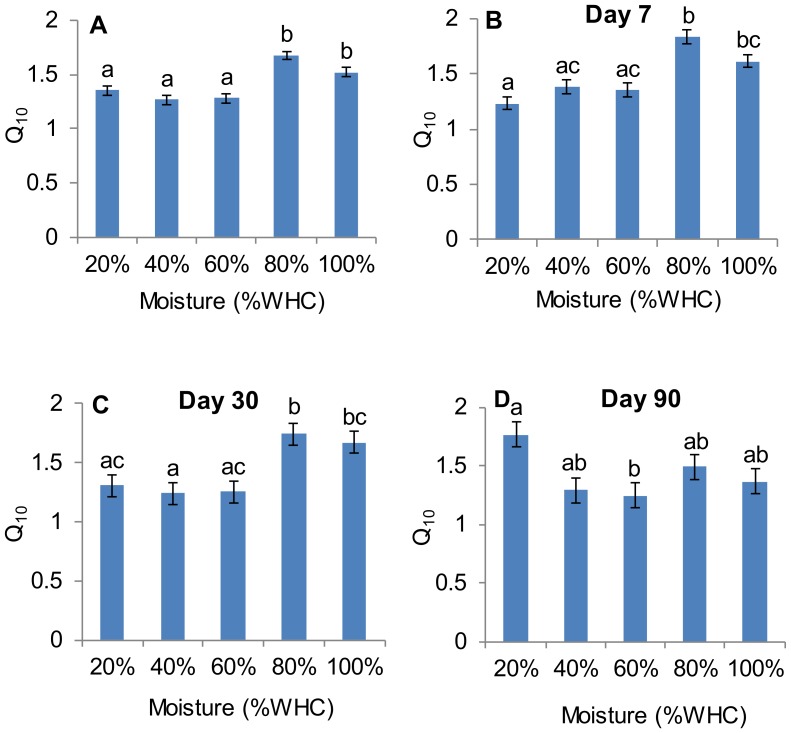
Comparisons of mean Q_10_ under different soil moisture treatments. The mean Q_10_ in panel (A) is calculated from 4 replicates across 15 measurement times (n = 60), and the mean Q_10_s in panel (B), (C) and (D) are calculated from 4 replicates on day 7, day 30 and day 90 (n = 4), respectively. Error bars represent standard errors. Lower case letters represent significant difference at P≤0.05.

Repeated-measures ANOVA showed that Q_10_ was significantly influenced by soil moisture (F _(4, 15)_ = 18.41, P<0.01), incubation time (F _(14, 210)_ = 15.41, P<0.01) and the interaction of the two (F _(56, 210)_ = 4.44, P<0.01). Averaged over the 15 measurement times, Q_10_ was significantly lower at 60%, 40% and 20% WHC compared to those at 80% and 100% WHC (Fig. 3A). Q_10_ at 80% WHC had the highest value but was not significantly different from that at 100% WHC (Fig. 3A).

Since the interactive effect of soil moisture and incubation time was significant, we further compared Q_10_ values among soil moisture treatments on three typical measurement days (Fig. 3B–D). On days 7 and 30, Q_10_ at 80% WHC was not significantly different from that at 100% WHC but significantly higher than those at the three lower moisture levels, which is similar to the all-day average results shown in Fig. 3A. On day 90, Q_10_ at 20% WHC was significantly higher than at 60% WHC, but there was no significant difference among the other three soil moisture treatments.

### Correlations between Q_10_ and Soil Properties

**Figure 4:**
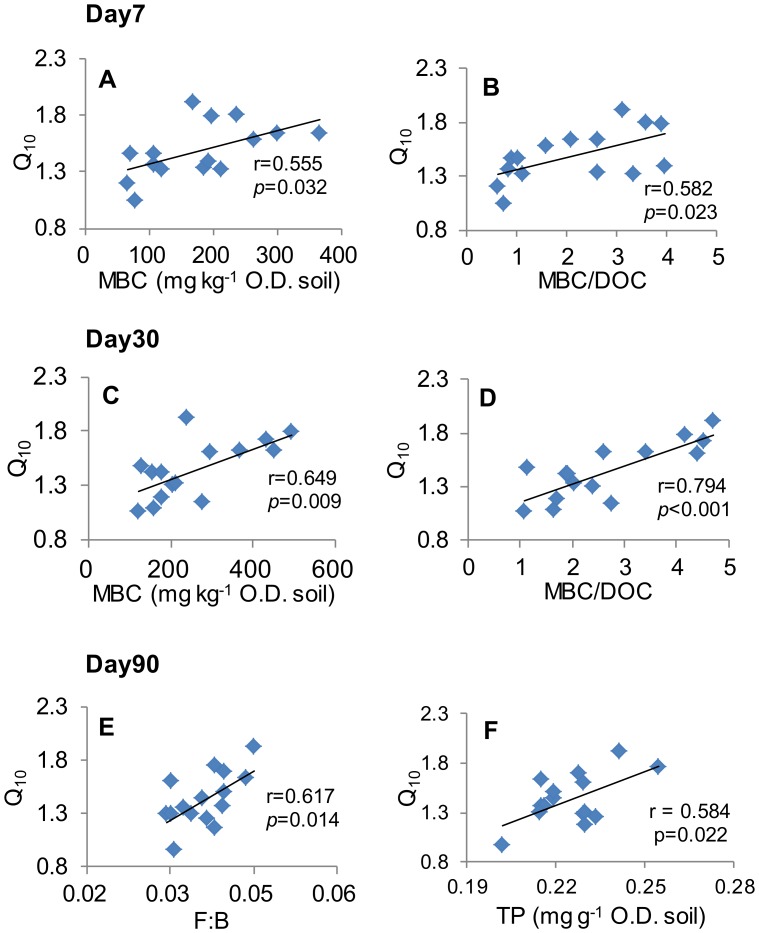
Correlations between Q_10_ and soil microbial and chemical properties. MBC: microbial biomass carbon, DOC: dissolved organic carbon, TP: total phosphorus, and F:B: ratio of fungi to bacteria. r is the correlation coefficient. P is the significance level.

Pearson correlation analysis showed that on days 7 and 30, Q_10_ was positively correlated with MBC and the ratio of MBC to DOC (Fig. 4A–D). On day 90, no significant correlation was found between Q_10_ and MBC or MBC/DOC; instead, Q_10_ positively correlated with the F:B ratio (Fig. 4E) and TP (Fig. 4F). We did not find significant correlations between Q_10_ and other soil chemical properties (inorganic nitrogen, DOC, TOC, and TN), which was therefore not presented.

## Discussion

### Moisture Effects on R_h_ Responses to Temperature

Similar to many previous results [2], [36], [37], soil R_h_ increased with temperature exponentially in our study; however response curves varied among different soil moisture treatments (Fig. 1, Table 2). Some previous studies showed that soil respiration may be decoupled from temperature under certain soil moisture levels resulting in soil respiration that is unaffected by temperature under water stress [38]. For example, Yu et al. [39] found that temperature was the determinant factor and R_h_ increased with it exponentially only when soil moisture was not limited. However, our results showed that soil R_h_ could still increase with temperature, even at 20% WHC, though at a relatively slow rate (Table 2). The discrepancy between our results and theirs may be because the studied soils differ in chemical, physical and microbial properties.

Regardless of temperature variations, soil R_h_ at 60% WHC tended to be higher than at both lower and higher soil moisture treatments. This result is also consistent with some other studies that reported higher R_h_ at intermediate moisture content [40]–[42]. The decrease of soil R_h_ at lower soil moisture has been attributed to soluble substrate limitation, whereas at higher soil moisture level, especially at saturated soil moisture, R_h_ was mainly limited by oxygen [12], [36]. The decrease of soil R_h_ over incubation time was probably caused by the depletion of labile substrate [43], [44]. However, the persistently high R_h_ at 60% WHC during the 90-day incubation could be due to the persistently high microbial activity, because this was the optimal moisture level and high microbial activity might override the influence of substrate limitation.

### Variations of Q_10_ with Incubation Time

The variation of Q_10_ with time has been found not to be uniform [45]. A laboratory incubation study found that Q_10_ increased with incubation time, which was ascribed to substrate quality change from labile to recalcitrant [46]. In some long-term warming experiments, Q_10_ was found to decline over time [1], [45]. In our relatively short-term incubation experiment, we found that Q_10_ declined with incubation time initially but increased during later incubation stages, and quadratic or cubic regression models were fitted to quantify the changes of Q_10_ at different soil moisture treatments (Fig. 2A). Over the 90-day incubation period, mean Q_10_ values was mostly <2.0, which is lower than the conventional estimates (2.0–2.6) probably due to less confounding factors involved in our incubation experiment [47]. The changes of Q_10_ might be related to the changes of soil R_h_, as many laboratory studies have shown that soil R_h_ decreases with incubation time [48]. The underlying mechanisms were ascribed to substrate depletion [43], [44]: the longer the incubation time, the more time microbes had to consume the labile carbon, leaving less to remain in the soil. In the absence of labile carbon, microbial mediated soil R_h_ tends to have lower Q_10_ [49], [50]. Similar variation patterns of Q_10_ with incubation time have been observed by Tuomi et al. [51] and Hamdi et al. [52], in which quadratic and cubic functions were also used to describe the relationships between Q_10_ and incubation time.

The increases of Q_10_ at the later stage might be related to soil substrate quality changes (Fig. 2A). As the labile carbon decreased, recalcitrant carbon could be decomposed. It has been previously reported that Q_10_ tends to be higher in this situation [53], [54]. In this study, it was not clear what caused the higher Q_10_ at 20% WHC at the later stage, but NH_4_^+^-N and TP were also higher at 20% WHC, which may be related to the higher Q_10_.

### Moisture Effects on Q_10_

We found that soil moisture had a significant effect on Q_10_, which aligns with the findings from several previous studies [17], [20], [55]. Our results showed that at the intermediate soil moisture level (i.e. 60% WHC), Q_10_ was lower than at the other soil moisture levels. While there was no significant difference of Q_10_ among 60%, 40% and 20% WHC, Q_10_ at 60% WHC was significantly lower than at 80% and 100% WHC (Fig. 3). Previous studies have shown that drying can decrease Q_10_ of soil respiration and total ecosystem respiration [2], [56], and this may be largely due to substrate limitation caused by the limited diffusion of solutes in thin soil water films [57], [58].

We further tested which soil properties would influence Q_10_ at different incubation days and found that, at the early and middle incubation stages, Q_10_ had a significant positive correlation with MBC and the ratio of MBC to DOC (Fig. 4). The higher MBC and MBC to DOC ratio were particularly associated with higher soil moisture levels, under which labile substrate might be more available to microbes due to less water limitation. However, the Arrhenius equation shows that reactants with lower activation energies (i.e. more reactive and less recalcitrant) should have lower temperature sensitivity [2]. Our incubation results indicated that Q_10_ might not only be determined by substrate availability, but also by microbial properties such microbial biomass.

At the late stage of incubation, Q_10_ was significantly related to F:B and TP. The tight correlation of Q_10_ with F:B ratio was quite interesting. Both fungi and bacteria are important decomposers, but their structures and chemical compositions are very different. Fungi have hyphae that allow them to move, colonize and degrade surface litters, and fungal cell walls are the polymers of melanin and of chitin, much more resistant to degradation [59], [60]. At the late stage, labile substrate diminishing may favor fungi communities which can degrade more recalcitrant substrate. As suggested by the carbon quality hypothesis [2], [61], soils with more fungi or higher F:B ratio would have larger Q_10_, as demonstrated here. Bradford et al. [62] also reported a shift in microbial community structure could alter the Q_10_ of R_h_. The positive correlation between Q_10_ and TP suggested that P availability might also influence Q_10_. For example, the Q_10_ value for the 20% WHC was higher than those for the other moisture levels (Fig. 2A) and TP was correspondingly higher, probably due to the lower rate of consumption by microbes at lower moisture levels. A field study also showed that summer drought caused a 22–64% reduction of microbial phosphorus [63], indicating lower microbial consumption of P under water stress. Furthermore, forest soils in subtropical China are often phosphorous limited [64]. The phosphorous saved by the lower rate of consumption might therefore contribute to the higher Q_10_ at 20%WHC during the late stage of the incubation.

## Conclusions

By incubating a subtropical forest soil under five temperature levels and five moisture levels and measuring soil R_h_ and microbial and chemical properties throughout the incubation, we found that: 1) soil moisture significantly influenced Q_10_, with Q_10_ being higher at higher soil moisture levels than at the lower moisture levels during the early stage of the incubation;2) soil heterotrophic respiration was highest at intermediate moisture and lowest when the soil was very dry; 3) Q_10_ mostly declined with incubation time and could be best described by quadratic or cubic functions; and 4) moisture-induced Q_10_ changes were associated with soil microbial biomass at the early stage of incubation, but to the ratio of fungi-to-bacteria at the late stage. These results imply that the response of soil R_h_ to future global warming may be shaped by changes in precipitation patterns. In dry conditions, global warming may stimulate less soil CO_2_ emission, but in wet conditions, relatively more soil CO_2_ may be emitted. Considering that more soil organic carbon has often been accumulated in the wet areas, with the same temperature rise high Q_10_ would mean more soil CO_2_ emission to the atmosphere from these areas in the future.
